# Physiological Investigation and Transcriptome Analysis of Polyethylene Glycol (PEG)-Induced Dehydration Stress in Cassava

**DOI:** 10.3390/ijms17030283

**Published:** 2016-02-25

**Authors:** Lili Fu, Zehong Ding, Bingying Han, Wei Hu, Yajun Li, Jiaming Zhang

**Affiliations:** Institute of Tropical Bioscience and Biotechnology, Chinese Academy of Tropical Agricultural Sciences, Xueyuan Road 4, Haikou 571101, China; fulili@itbb.org.cn (L.F.); dingzehong@itbb.org.cn (Z.D.); hanbingying@itbb.org.cn (B.H.); huwei2013@itbb.org.cn (W.H.); liyajun@itbb.org.cn (Y.L.)

**Keywords:** cassava, abiotic stress, transcriptome analysis, RNA-seq, abscisic acid

## Abstract

Cassava is an important tropical and sub-tropical root crop that is adapted to drought environment. However, severe drought stress significantly influences biomass accumulation and starchy root production. The mechanism underlying drought-tolerance remains obscure in cassava. In this study, changes of physiological characters and gene transcriptome profiles were investigated under dehydration stress simulated by polyethylene glycol (PEG) treatments. Five traits, including peroxidase (POD) activity, proline content, malondialdehyde (MDA), soluble sugar and soluble protein, were all dramatically induced in response to PEG treatment. RNA-seq analysis revealed a gradient decrease of differentially expressed (DE) gene number in tissues from bottom to top of a plant, suggesting that cassava root has a quicker response and more induced/depressed DE genes than leaves in response to drought. Overall, dynamic changes of gene expression profiles in cassava root and leaves were uncovered: genes related to glycolysis, abscisic acid and ethylene biosynthesis, lipid metabolism, protein degradation, and second metabolism of flavonoids were significantly induced, while genes associated with cell cycle/organization, cell wall synthesis and degradation, DNA synthesis and chromatin structure, protein synthesis, light reaction of photosynthesis, gibberelin pathways and abiotic stress were greatly depressed. Finally, novel pathways in ABA-dependent and ABA-independent regulatory networks underlying PEG-induced dehydration response in cassava were detected, and the RNA-Seq results of a subset of fifteen genes were confirmed by real-time PCR. The findings will improve our understanding of the mechanism related to dehydration stress-tolerance in cassava and will provide useful candidate genes for breeding of cassava varieties better adapted to drought environment.

## 1. Introduction

Cassava (*Manihot esculenta* Crantz) is an important cash crop for many poor farmers in marginal areas of tropics and sub-tropics regions [1,2]. As a food security crop, it provides nourishment for over 750 million people around the world [1]. Besides, cassava is considered as one of the major crops for starch, bio-fuel production, and animal feed due to its starch-enriched root [3]. Cassava is generally tolerant to drought, however, similarly to other crops, drought stress greatly influences many physiological processes of cassava and depresses its growth, development and economic yield [3,4]. Thus, it is of great importance to increase our understanding of the mechanisms underlying cassava tolerance to drought stress.

During the long process of adaptation and domestication, plants have developed different physiological and biochemical strategies in response to drought stress. When the leaf-to-air vapor pressure or relative humidity changes, one of the most rapid responses is that plants will quickly close its stomata to protect the leaf against water loss and to maintain high water use efficiency [5,6]. When suffering prolonged and progressive drought conditions, canopy photosynthesis of plants is significantly reduced [7]. To adapt to water shortage, plants will decrease their leaf canopy to reduce water use or increase their root length to get access to deep water layers [3,6]. Meanwhile, various small molecule compounds such as soluble sugars, soluble protein and proline are synthesized and accumulated to maintain water content of cells [8,9]. When plants were grown under severe drought stress, reactive oxygen species (ROS), which have detrimental effects on plant growth and development, is produced. To deal with oxidative damage, several enzymes including superoxide dismutase (SOD), peroxidase (POD), and catalase (CAT) are activated in plant cells [9,10,11]. As one of the end products of lipid peroxidation, malondialdehyde (MDA) content has been considered an indicator of oxidative damage, reflecting the extent of the peroxidation of membrane lipids and the tolerance of plants towards stress conditions [12]. However, the dynamic changes of these molecular compounds and enzyme activities were largely unknown in response to drought stress in cassava.

Hormones and transcription factors (TFs) are key regulators that are involved in plant drought stress signaling [13,14]. Under drought condition, abscisic acid (ABA) levels were strongly increased in plants, accompanied by dramatic changes (either induced or depressed) of the expression levels of many genes [15,16]. Although ABA is the best known hormone messenger triggering the cascade of drought signaling, it is worth noting that other hormones such as ethylene, jasmonates (JA), and cytokinin (CK) were also involved in the stress response [17]. AREB/ABFs are bZIP transcriptional factors (TFs) that regulate ABA-responsive gene expression [18]. Totally, nine AREB/ABF members were identified in Arabidopsis, of which *AREB1/ABF2*, *AREB2/ABF4*, and *ABF3* were highly induced by ABA and they cooperatively regulated ABRE-dependent ABA signaling in drought stress tolerance [19]. Arabidopsis transgenic plants over-expressing *AREB1* showed enhanced drought tolerance and ABA hypersensitivity [20]. Similar conclusions were observed in rice and soybean [21,22]. Besides AREB/ABFs, TFs such as WRKY [23], NAC [24], AP2 [25], and MYB [26] were also involved in plant drought stress response. However, these results were mainly derived from model species (e.g., Arabidopsis), far less was known about tropical species such as cassava.

In the past two decades, much progress has been made in large-scale screens for genes of particular traits in cassava. For example, cDNA and oligonucleotide microarray were used to identify differentially expressed genes that were associated with post-harvest physiological deterioration [27], bacterial blight disease [28], storage roots development [29], cold [30] and drought [1] stresses. iTRAQ-based proteomic analysis was also employed to study different strategies of cassava in response to drought stress [31]. As the rapid improvement of next generation sequencing techniques and the release of cassava draft genome [32,33], RNA-seq has become a new way to study the gene expression on a global level in cassava research [34,35,36]. However, there are very few RNA-seq analyses concerning drought stress in cassava, and the mechanism underlying cassava drought-tolerance remains obscure, and thus needs to be further explored.

In this study, we report our findings on physiological changes of cassava leaves in response to different levels of dehydration stress simulated by polyethylene glycol (PEG), which is commonly used when evaluating plant drought tolerance. Subsequently, RNA-seq was employed to investigate the dynamic changes of gene expression profiles in cassava leaves (at different developmental stages) and root. The results will provide new insights in the regulation networks of dehydration stress in cassava and the genetic improvement of cassava varieties better adapted to drought environment.

## 2. Results

### 2.1. Physiological Changes of Cassava Leaves

**Figure 1:**
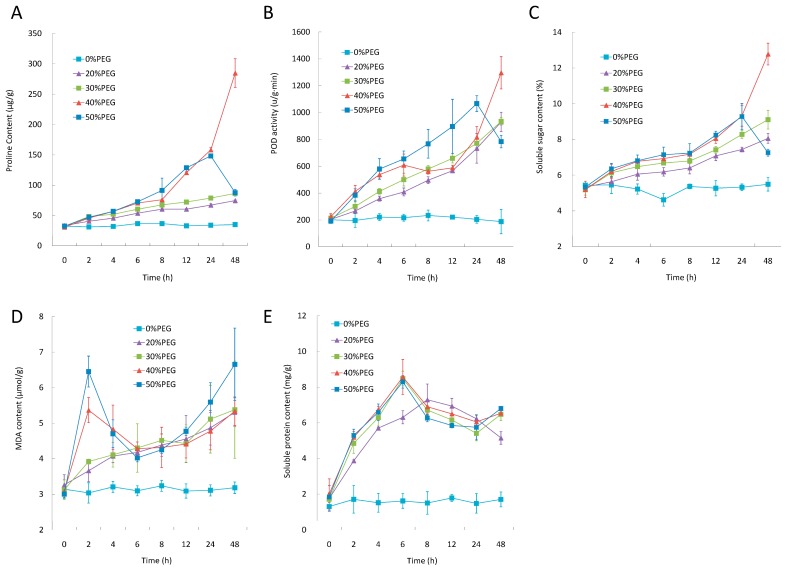
Physiological changes of cassava leaves in response to PEG treatments. Proline content (**A**); POD activity (**B**); soluble sugar content (**C**); MDA content (**D**); and soluble protein content (**E**) were investigated under different PEG concentrations (0%, 20%, 30%, 40% and 50%) across eight time points (0, 2, 4, 6, 8, 12, 24 and 48 h) within 48 h.

In order to investigate the dynamic changes of cassava in response to different PEG treatments, five physiological traits, including proline, MDA, soluble sugar and soluble protein content, and POD activity that play important roles under drought stress to either maintain water content or reduce oxidative damage in plant cells, were measured under five PEG concentrations (0%, 20%, 30%, 40% and 50%) across eight time points (0, 2, 4, 6, 8, 12, 24 and 48 h) in leaves (Figure 1 and Figure S1 (Supplementary Material)).

Overall, five traits were almost unchanged across different time points under control condition (0% PEG). The contents/activities were quickly increased when PEG was applied in all concentrations (Figure 1).

Interestingly, proline content, POD activity, and soluble sugar content consistently showed strict gradient changes: steady linear increases in contents and/or activities were observed under both 20% and 30% PEG treatments over time, while there was a sudden and sharp increase under 40% PEG but decrease under 50% PEG treatment, respectively, at the time point from 24 to 48 h (Figure 1A–C). These results suggest that proline content, POD activity, and soluble sugar content were highly correlated with each other in response to PEG treatments and that high concentrations (e.g., 50%) of PEG plus prolonged (e.g., >24 h) treatment destroyed the plant’s ability to adjust its cell osmotic status.

Similarly, MDA content also exhibited a linear change under 20% and 30% PEG treatments, but it showed a high peak at 2 h when 40% and 50% PEG treatments were applied (Figure 1D). Soluble protein content also showed steady increase during the first 6 to 8 h, however, decreased in prolonged osmotic stress at all PEG concentrations, and the turning point came earlier when plants received higher concentrations (30%–50%) of PEG treatments (Figure 1E).

The dramatic physiological changes indicate that PEG treatment has great effect on cassava leaves similar to that caused by drought stress in other crops [8,9]. To further explore the mechanism of dehydration stress stimulated by PEG, RNA-seq was employed to investigate the changes of genome-wide gene expression in folded leaf (FL), full expanded leaf (FEL), bottom leaf (BL), as well as root (RT) in cassava.

### 2.2. Profiling of Cassava Leaf Transcriptome

In total, 146 million raw reads of 49-bp length were generated by single end sequencing with Illumina HiSeq 2000 machine. After trimming adapters and filtering out low quality reads, approximately 143 million clean reads were obtained, 88.3% of them were aligned to the cassava genome and were used for further analysis.

To minimize the false positive of expressed genes, a threshold cutoff, CPM (Counts Per Million mapped reads) >10, was arbitrarily used to identify genes that were expressed among samples. In total, 18,886 genes, equal to about two thirds of the annotated genes in the genome (phytozome Mesculenta version 4.1), were expressed across all 12 samples.

**Figure 2:**
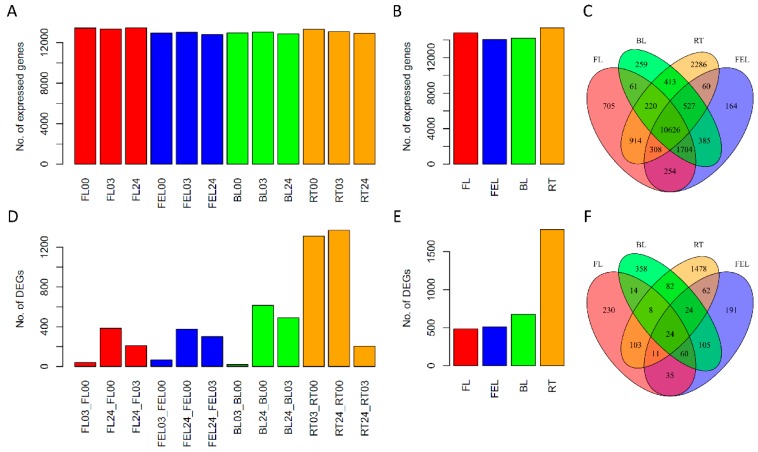
Transcriptome profiling of cassava in response to PEG stress. Expressed genes identified in 12 samples (**A**); four tissues (**B**) and their Venn diagrams (**C**); respectively; differentially expressed (DE) genes identified in 12 samples (**D**); four tissues (**E**); and their Venn diagrams (**F**), respectively. FL: folded leaf; FEL: full expanded leaf; BL: bottom leaf; RT: root. The numbers attached behind leaf samples represent the time point at which samples were collected: e.g., 00, 03, and 24 represent 0, 3, and 24 h, respectively.

Overall, quite similar numbers of expressed genes were observed in all samples of FL, FEL, BL and RT across three time points (0, 3, 24 h) (Figure 2A). The numbers rose a bit but were still almost equivalent among these four tissues (Figure 2B) when considering the three time points together (which means a gene was counted if it was expressed in at least one out of three time points of the same tissue). Accordingly, most (10,626) of these genes were expressed in all tissues, while only a small amount of genes were exclusively expressed in FL (705), FEL (164), BL (259), and RT (2286) (Figure 2C).

To confirm the RNA-seq results, 15 drought-induced genes that are involved in the ABA-dependent and ABA-independent pathways [37] were selected and their expression patterns were validated by qRT-PCR with high correlation coefficients (*R* = 0.75–0.93) between the two technologies (Table S1A,B, Supplementary Material).

### 2.3. Transcriptome Changes Triggered by PEG Stress

To reveal the transcriptome changes influenced by PEG treatment, differentially expressed (DE) genes that had both relatively high expression levels (e.g., CPM ≥ 10) and high expression changes (e.g., FC ≥ 3) were identified by pair-wise comparison of samples collected at different time points within the same tissue, respectively. Overall, in the leaf samples, only a few genes were differentially expressed after 3 h of PEG treatment, but the DE gene numbers were greatly increased after 24 h treatment. In contrast, this tendency was quite different in root: a large number of genes were differentially expressed after 3 h treatment, while only a few gene expression levels were further changed at 24 h compared with 3 h treatment (Figure 2D). Moreover, we observed a gradient change of DE gene number from bottom to top of a plant: RT (1792) > BL (675) > FEL (512) > FL (485) (Figure 2E). These results suggested that root had a quicker response and more induced DE genes than leaves in response to PEG stress. In contrast to expressed genes, most of DE genes were exclusively identified in RT (1478), FL (230), FEL (191) and BL (358), and only 24 were identified in all four tissues (Figure 2F).

**Figure 3:**
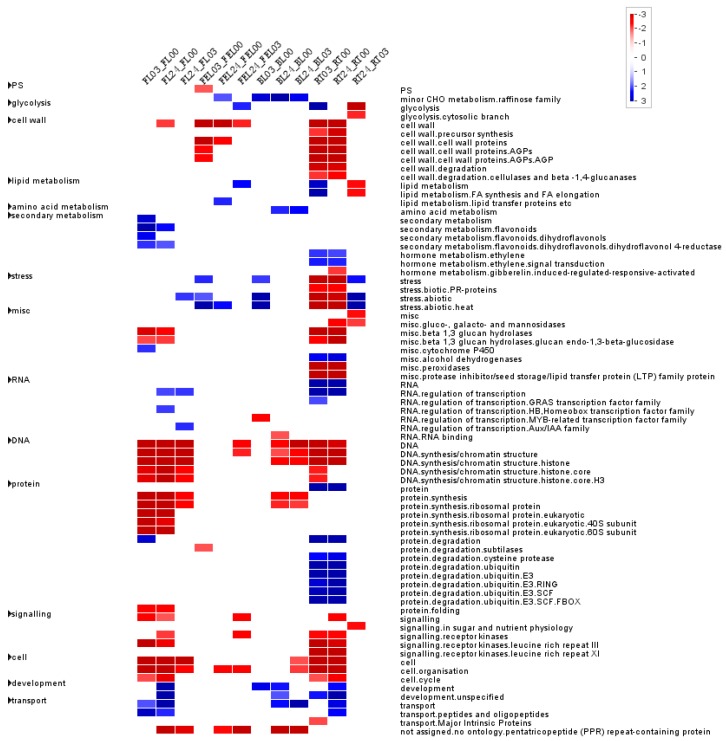
Functional category enrichment of DEGs by pair-wise comparison. Each column represents the DE genes that are dramatically up (**blue**) or down (**red**) regulated when comparing two samples indicated at the top, while each row represents a functional category derived from the MapMan software. Enriched functional categories were identified based on Wilcoxon test that embedded in PageMan where ORA cutoff value was set to 1 and *p*-values were Benjamini-Hochberg corrected.

To investigate the functional pathways in which DE genes were influenced, PageMan was used to perform functional categories enrichment of DE genes by pair-wise comparison. As shown in Figure 3, genes related to secondary metabolism, RNA regulation of transcription, and transport were significantly up-regulated, while genes related to DNA synthesis/chromation structure, protein synthesis, signaling and cell-related metabolisms were significantly down-regulated in FL. The regulation tendency was similar in RT, including RNA regulation of transcription, transport, DNA synthesis/chromation structure, signaling and cell-related metabolisms, but with a few exceptions: e.g., categories such as protein degradation and ethylene metabolism were up-regulated, while categories such as cell wall, abiotic stress and hormone gibberelin were down-regulated. In contrast to FL and RT, far fewer categories were enriched in FEL and BL (Figure 3).

### 2.4. Functional Category and DE Gene Clustering

**Figure 4:**
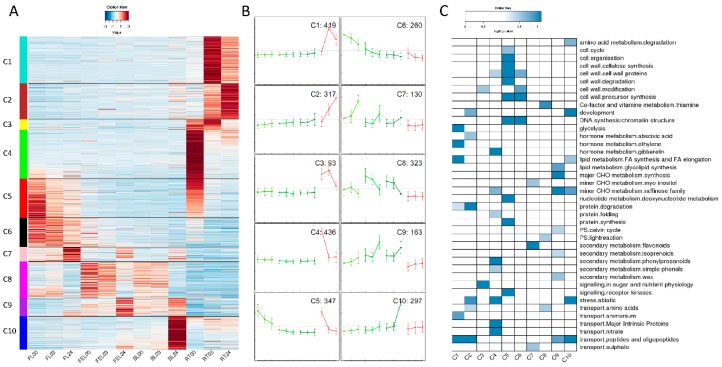
Gene expression patterns across leaf and root samples. (**A**) Heatmap of 2785 DEGs that were grouped into ten clusters by K-mean method; (**B**) Expression patterns of ten clusters in (**A**). The samples (from **left** to **right**) are the same as those presented in (**A**). Error bars represent standard deviation, and DEG numbers are shown in the upper right corner; (**C**) Functional category enrichment of each cluster in (**A**).

To investigate the biological function of DE genes in response to PEG stress, a total of 2785 genes (about 9% of annotated genes in the cassava genome), which were significantly differentially expressed in at least one pair-wise comparison, were subjected to hierarchical clustering based on Pearson correlation. A total of 10 clusters (C1–C10, Figure 4A,B) were identified based on their expression patterns. Functional category enrichment analysis was performed for each cluster to identify the common and different pathways in the particular categories.

As shown in Figure 4A,B, C1 to C4 clusters included genes that had low expression in all leaf samples but high expression in RT, in which the differences of these clusters were presented. The genes in the C1 cluster were greatly induced from 0 to 3 h, but decreased at 24 h in RT, and they were enriched in glycolysis, ethylene, FA synthesis and elongation, ammonium as well as peptides transport (Figure 4C). The genes in the C2 cluster were also significantly induced in RT, but their highest expression level came at 24 h. These genes were enriched in development, abscisic acid, protein degradation, abiotic stress and peptides transport. In contrast to C1 and C2 clusters, the genes in both C3 and C4 clusters were drastically depressed. The enriched categories included cell wall, gibberelin, minor CHO metabolism of raffinose family, secondary metabolism, signaling in sugar, abiotic stress, and transport of nitrate and major intrinsic proteins (Figure 4C).

Compared with the C4 cluster, genes in the C5 cluster showed a similar expression tendency in FEL, BL, and RT, but their expression level was gradually decreased in FL in response to PEG treatment (Figure 4A,B). The greatest enrichment of this cluster was observed in cell cycle, cell organization, multiple cell wall-related pathways, DNA synthesis/chromatin structure, nucleotide metabolism, protein synthesis and receptor kinases signaling (Figure 4C).

C6 to C10 clusters included genes that had low expression in RT but high expression in leaf samples, and the differences of these clusters were only presented in leaf. The genes in C6 and C8 clusters were greatly depressed in leaf after PEG treatments; the difference is that the genes in C6 were highly expressed in FL while the genes in C8 were highly expressed in both FEL and BL (Figure 4A,B). The enriched categories included cell wall, DNA synthesis/chromatin structure, light reaction of photosynthesis and amino acids transport (Figure 4C). Similarly to C6 and C8 clusters, the genes in C7 were highly expressed in FL while the genes in C9 were highly expressed in FEL and BL. However, the genes in C7 and C9 clusters were both greatly induced by PEG treatment (Figure 4A,B). The enriched categories included secondary metabolism, glycolipid synthesis, major and minor CHO metabolism, calvin cycle, sulphate and peptides transport (Figure 4C). Unlike previous clusters, the genes in the C10 cluster showed highest expression in BL after 24 h of PEG treatment. These genes were enriched in amino acid degradation metabolism, development, FA synthesis and elongation, minor CHO metabolism of raffinose family, abiotic stress and peptides transport.

Taken together, the DE genes in C1 to C10 clusters revealed that the major biochemical shifts among samples (e.g., leaf *vs.* root) were produced in part by highly dynamic, coordinated and localized transitions in mRNA abundance.

### 2.5. Responses of Abiotic Stress-Related Genes

After manual curation, a total of 92 abiotic stress genes were differentially expressed in response to PEG treatment. The most dominant category was related to heat stress (75 genes), followed by drought/salt (nine genes), touch/wounding (five genes) and cold stress (three genes, Table S2, Supplementary Material).

Hierarchical clustering grouped these genes into four main clusters (Figure S2, Supplementary Material). Genes of cluster S1 (60%, 55 genes) and S4 (13%, 12 genes) are highly expressed at RT; the former was dramatically depressed while the later induced in response to PEG treatment. As comparison, the gene expression of cluster S2 (11%, 10 genes) was significantly decreased in both FEL and BL, while cluster S3 (16%, 15 genes) was greatly induced in BL, especially after 24 h of PEG treatment. These results indicated that abiotic stress genes have different responses to PEG treatment and more genes were differentially expressed in RT than in leaves (67 *vs.* 25).

### 2.6. Responses of Hormone-Related Genes

In plants, hormones usually play central roles in a diverse set of developmental processes as well as changing environments. In this study, in total, 92 hormone related genes (Table S3, Supplementary Material), referred to eight hormones including abscisic acid (ABA), auxin, brassinosteroid (BR), cytokinin (CK), ethylene, gibberellic acid (GA), jasmonic acid (JA) and salicylic acid (SA), were differentially expressed in response to PEG treatment. The three hormones with most abundant genes were ethylene (32), auxin (19), and GA (16). As expected, ethylene and GA related genes were significantly enriched. Genes related to another hormone, ABA, was also enriched (Figure 4C). Although the three hormone-related genes were highly expressed in RT, their expression patterns were quite different (Figure 4A). For example, genes related to ABA, including ABA biosynthesis gene *NCED3* and ABA signal transduction gene *ABF2*, were gradually increased from 0 to 3 h, and reached their highest expression levels at 24 h; ethylene associated genes, including ACC oxidase 1 (*ACO1*) and ethylene-forming enzyme (*EFE*) that catalyze the final step of ethylene biosynthesis, were greatly induced from 0 to 3 h in RT, but their expression levels were decreased at 24 h; while GA related genes such as GAST1 protein homolog 4 (*GASA4*), were expressed highly at 0 h but expression sharply decreased at both 3 and 24 h (Table S3, Supplementary Material). These results suggested that ABA, ethylene, and GA are the key hormones involved in drought adaptation in cassava.

### 2.7. Responses of Transcription Factors (TFs)

**Figure 5:**
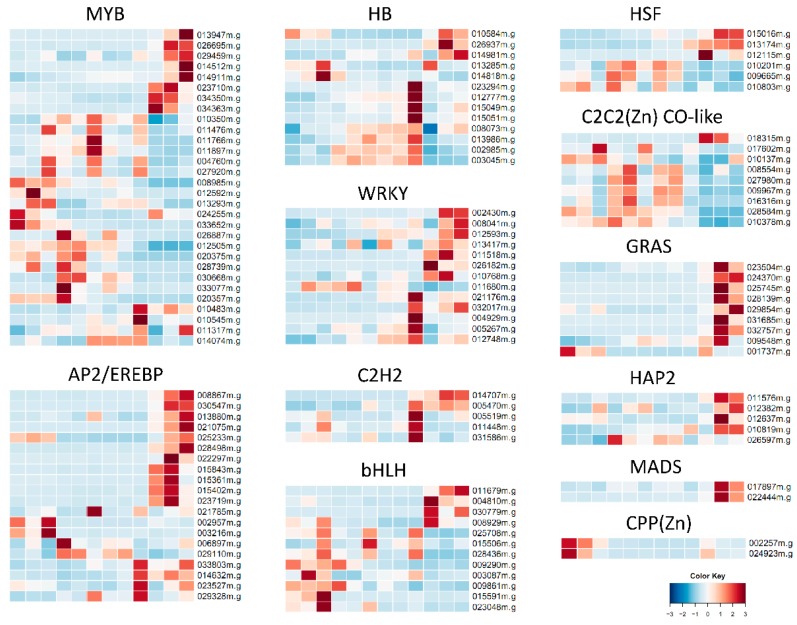
Expression pattern of TFs in cassava leaves and root in response to PEG stress. The samples (from **left** to **right**) in heatmaps were the same as those in Figure 4A.

In total, 1179 TF genes were expressed in at least one sample of our study, and 170 (14.4%, representing 25 families) of them were differentially expressed in response to PEG treatment (Table S4, Supplementary Material). The four most abundant TF families were MYB (30), AP2/EREBP (20), HB (13), and WRKY (13), followed by bHLH (12), C2C2(Zn) CO-like (9), and GRAS (9, Table S4, Supplementary Material). We inspected the expression level of genes within each TF family, respectively, and different expression patterns were observed. For example, MYB family genes showed multiple diverse expression patterns (Figure 5), indicating that these members had different responses to PEG stress. In comparison, most members of AP2/EREBP, HB, WRKY, and C2H2 were highly expressed either in RT or in BL after 24 h of PEG treatment, bHLH was high expressed in either RT or FL, while HSF was highly expressed in either RT or FEL/BL. The majority of members of C2C2(Zn) CO-like were highly expressed in both FEL and BL, but their expression was greatly decreased at 24 h after PEG treatment. It is worthy to note that most members of GRAS, HAP2 and MADS were highly induced only in RT, while those of CPP(Zn) were exclusively depressed only in FL, in response to PEG treatment (Figure 5).

Based on K-means clustering, 170 TFs were well segregated into six groups according to their expression patterns (Figure S3, Supplementary Material). There were 45 genes in G1 and 21 in G2 clusters. Although they were both higher expressed in root than in leaf, the expression of genes were greatly induced in G1 but depressed in G2 clusters (Figure S3, Supplementary Material). Among them, multiple TF families involved in hormones were identified, e.g., HB family (*HB7*) for ABA, AP2/EREBP family (*RAP2.11*) for ethylene, GRAS family (*SCL3*, *RGL1*, *RGL2*) for GA and WRKY family (*WRKY70*) for both JA and SA in G1 cluster; Aux/IAA family (*IAA7*, *IAA9*) and MYB family (*MYB77*) for auxin, AP2/EREBP family (*CBF3*, *CBF4*) for ABA in G2 cluster (Table S4, Supplementary Material).

There were 17 genes in the G3 cluster. Their expression was dramatically increased in all three leaf tissues at 24 h after PEG treatment. Several TFs associated with light signaling (*PIL5*) and circadian rhythm (*LHY*, *APRR5*) were included in this cluster (Table S4, Supplementary Material).

There were 27 genes in the G4 cluster, and these genes were highly expressed in FL. Many TFs related to development were included in this cluster: e.g., leaf differentiation (*TCP3*), embryo axis formation and vascular development (*MP*), cell proliferation (*ANT*), chloroplast development (*GPRI1*), and trichome branching (*MYB5*). In addition, TFs involved in anthocyanin biosynthesis (*TT8*), phenylpropanoid biosynthesis (*MYB3*) and light signaling (*CIB1*) were also included (Table S4, Supplementary Material).

Compared with the G3 cluster, the expression of genes in G5 (31 genes) were greatly depressed in FEL and BL. Similarly, circadian rhythm associated TFs (*PCL1*, *APRR5*) were included in this cluster. Besides, TFs related to leaf differentiation (*TCP4*), heat shock (*HSFA8*, *HSFA6B*, *HSFC1*), as well as putative drought response (*DRE2B*) were included.

In total, there were 29 genes in the G6 cluster. The expression of these genes was dramatically induced only in BL after 24 h of PEG treatment. TF genes that associated with leaf and hypocotyl development (*HB1*), freezing stress (*CEJ1*), and both drought and ABA signaling (*HB7*) were included in this cluster.

Taken together, the diverse expression patterns of TFs revealed that they were involved in different functions in response to PEG stress, e.g., G1 and G2 clusters mainly for hormone metabolism; G3 and G5 clusters for light signaling and circadian rhythm; and G4 cluster for plant development.

## 3. Discussion

### 3.1. Physiological Responses of Cassava to Drought Stress

Drought is one of the most universal stresses influencing plant growth and crop productivity in the world. Under drought stress conditions, various small molecules such as proline, soluble sugars and soluble proteins that play important roles in osmotic adjustment are quickly accumulated [8,9]. Besides, antioxidative enzymes such as superoxide dismutase (SOD), peroxidase (POD) and catalase, as well as MDA content are induced quickly and required to deal with oxidative damage during the period of drought stress [9,10,11]. Thus, the content of these molecules and the activity of these enzymes are widely used as parameters to evaluate the characteristics of plants when they are suffering from drought [8,9,38]. Although cassava is a drought-tolerant crop, the physiological responses of cassava to drought stress have not been extensively explored.

In this study, five traits including POD activity, the content of proline, MDA, soluble sugar and soluble protein, were investigated in cassava leaves at eight time points (within two days) under several dehydration stresses simulated by different concentrations of PEG solutions. Similar to previous studies in other species [8,38], these five investigated traits revealed a rapid induction in response to PEG treatment. It is worth noting that, under low PEG concentration (e.g., 20% and 30%), POD activity and the content of proline, MDA and soluble sugar showed a linear increase as treatment time went up; even under high PEG concentration (e.g., 40% and 50%), three of them (except MDA content) still exhibited very consistent performance, indicating they played highly coordinate functions in cassava. Similar results were also found in other species [38,39]. We also noted that, when PEG concentration increased to 40% or 50%, the tendency of these parameters were quite different (e.g., break points were observed at 2 h for MDA content and at 24 h for POD activity, proline and soluble sugar content) comparing with those treated with 20% or 30% PEG, suggesting that the balance of cell osmotic adjustment was broken when plants suffered longer and more severe drought stress [38]. These results also demonstrated that PEG treatment is a good method to simulate drought stress in cassava studies and that the PEG concentration should not exceed 30% if monitoring the responses of cassava for a period longer than 24 h.

In general, phenotypes are often regulated by changes in gene expression. Here we found that the expression of one delta1-pyrroline-5-carboxylate synthase (*P5CS*, 002371m.g) which is a key enzyme in the synthesis of proline [40], and two POD genes (011604m.g and 023402m.g) was gradually increased in leaves with sustained stress. Their expression patterns coincided with the changes in physiological traits, indicating that these genes play important roles in drought stress of cassava.

### 3.2. Roles of Heat Shock Proteins in Drought

Heat shock proteins (Hsps) are widely distributed in plants and animals. Their transcripts are significantly influenced not only by heat stress, but also by others such as drought, salinity, cold, oxidative stress and wounding [41,42]. In total, 92 genes related to abiotic stress were differentially expressed in our study, 82% (75/92) of them were classified into the heat category containing 65 Hsps based on Mapman annotation, suggesting that Hsps play major roles in PEG-simulated dehydration stress in cassava.

It has been suggested that Hsps could act as molecular chaperones to regulate protein folding, localization and degradation [43]. In addition, Hsps could also protect proteins from damage to maintain the correct protein structure [44]. It seems that one of Hsps functions was related to protein folding. This hypothesis could be verified by gene co-expression analysis, which is one of the useful tools to predict functions of genes which have similar expression patterns [45]. In our study, we found that the expression of 71% (46/65) Hsps was dramatically depressed in response to PEG treatment and grouped into the C4 cluster in Figure 4A. As expected, we found that categories of both abiotic stress and protein folding were significantly enriched in this cluster (Figure 4C), supporting that the roles of Hsps are associated with protein folding. Besides, categories of cell wall proteins and GA, which is an important hormone to regulate plant growth and development, were also enriched in this cluster, confirming that cassava would minimize its metabolic activities (e.g., cell wall synthesis) to activate the “survival” model [31] in adaptation to drought environment.

It has been demonstrated that the transcripts of Hsps are regulated by heat stress transcription factors (Hsfs) and each Hsf has its role in regulatory networks in plants [42]. In total six Hsfs DE genes were found in this study, and only one (012115m.g) was co-expressed with Hsps in the C4 cluster (Figure 4A), suggesting that this Hsf is a key regulator of drought stress response in cassava.

The function of genes could be confirmed by different experimental comparison. By comparing our results with a recent proteomic study [31], we found that 18 Hsps were commonly found to be differentially expressed in response to drought. In addition, three of them (018200m.g, 018158m.g and 017871m.g) were included as the top 10 most strongly up- or down-regulated proteins [31]. Taken together, these results strongly suggest that Hsps play important roles in drought stress in cassava.

### 3.3. Regulatory Networks of Drought Stress in Cassava

**Figure 6:**
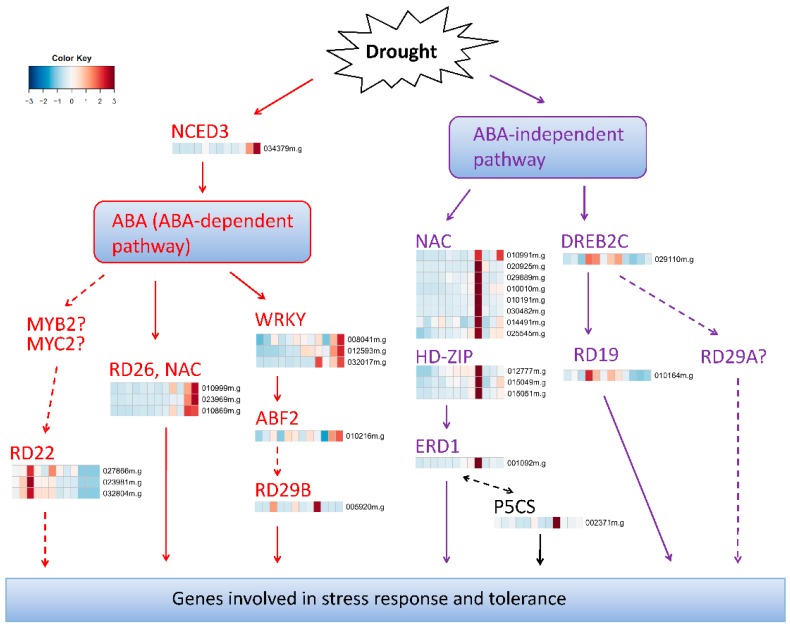
A model of transcriptional regulatory networks in response to drought stress in cassava. This figure is modified from Figure 2 in ShinozakiandYamaguchi–Shinozaki ShinozakiandYamaguchi–Shinozaki [46]. ABA-dependent and ABA-independent pathways were marked with red and purple, respectively. DE genes were mapped to the regulatory networks based on Mapman annotation and/or gene co-expression patterns. Solid lines indicate the interactions that were consistent with previous reported studies, while dash lines represent the interactions reported in other plants but not confirmed in our study. Question marks indicate the genes that were not differentially expressed and their functions need to be further validated in cassava. The samples (from **left** to **right**) indicated by cells in heatmaps were the same as those in Figure 4A.

It has been well demonstrated that there are two regulatory systems (ABA-dependent and ABA-independent) governing drought-inducible gene expression in plants [37,46]. These two regulatory systems could be further sub-divided into at least five signal transduction pathways including three ABA-dependent and two ABA independent paythways [46]. To reveal the different and common characteristics of drought response mechanisms between cassava and other plants, DE genes were mapped to these regulatory networks based on Mapman annotation and/or gene co-expression (Figure 6, Table S5 (Supplementary Material)).

*NCED3* (9-*cis*-epoxycarotenoid dioxygenase) is a key enzyme in ABA synthesis, its expression is strongly induced by dehydration and high salinity [37]. In our study, we observed that *NCED3* is expressed at very low levels in all tested normal tissues, but its expression was dramatically stimulated by PEG-treatment specifically in root (Figure 6). Several TFs such as NAC, bZIP, WRKY, AP2/EREBP, MYB and bHLH were reported to be involved in ABA-mediated drought stress [18,46]. *RD26* is a drought-inducible NAC transcription factor, and *RD26* over-expression trans-genetic plants were hypersensitive to ABA [47]. As expected, *RD26* showed very consistent expression patterns as *NCED3* across all tested samples. Besides, two novel NAC proteins, *NAC025* and *NAC100* (Figure 6), were also found, suggesting they might play similar roles as *RD26* in the drought environment. *ABF2* is a bZIP TF that binds to ABA-responsive element (ABRE) and controls ABA-responsive gene expression [48]. WRKY TFs such as *WRKY40* and *WRKY63* interact with *ABF2* and co-regulated ABA responses [23]. In this study, *ABF2* and three WRKY TFs (*WRKY1*, *WRKY21* and *WRKY23*) were differentially expressed in response to PEG treatments and showed consistent ABA-induced expression trends as *NCED3* (Figure 6), confirming that these ABA-dependent regulatory pathways also worked in cassava. However, *RD29B*, which was activated by *AREB1/ABF2* and involved in ABA signal pathways [46,49], showed quite different expression patterns (Figure 6). Besides, *RD22*, another drought inducible and ABA-mediated TF [46], also showed different expression patterns (Figure 6); in addition, two TFs of *MYC2* and *MYB2*, which could bind to the *cis*-element of *RD22* promoter and co-operatively active *RD22* [50], were not even differentially expressed in our study, suggesting that the functions of these genes were changed or they did not play important roles in ABA-dependent pathways of cassava.

Similar to the ABA-dependent pathway, TFs that were reported to be involved in the ABA-independent pathway were also confirmed in cassava. *ERD1*, which encodes a Clp protease regulatory subunit, was up-regulated by drought as well as natural and dark-induced senescence but not by ABA [37,51]. Using yeast one-hybrid screening, three NAC TFs including *NAC019*, *NAC055* and *NAC072* were found to bind to one of the *cis*-elements in the *ERD1* promoter [52]. Several HD-ZIP TFs like *HB7* and *HB12* were involved in water deficit response [18,53]. Here, we found that *NAC019*, *HB7* and *HB12*, along with several other NAC and HD-ZIP TFs, were co-expressed with *ERD1* (Figure 6). Although the expression of *HB7* and *HB12* were reported to be dependent on ABA [53], their expression patterns are very different with ABA biosynthesis gene (e.g., *NCED3*), suggesting that *HB7* and *HB12* may co-operate with *ERD1* [46] and other TFs [37] to play key roles in ABA-independent pathways in cassava. Some exceptions were also observed. Out of eight DREB2 genes in Arabidopsis, *DREB2A* and *DREB2B* function as transcriptional activators in ABA-independent pathway through *RD29A* [46,54]. Nevertheless, none of these three genes were found to be differentially expressed in our study. Instead, another DREB2 member, *DREB2C*, which was involved in freezing and heat tolerance and ABA-insensitive pathways [48], was found and co-expressed with a NAC protein, *RD19*, which was in response to dehydration and also not induced by ABA [55] (Figure 6). Interestingly, *P5CS*, which functions as a rate-limiting enzyme in proline biosynthesis [40], also exhibited consistent expression patterns with *ERD1* (Figure 6). This result is consistent with a very recently reported study [56], and it suggests that *P5CS* might participate in an ABA-independent pathway via *ERD1* in cassava.

Taken together, these results suggest that besides the common drought-tolerance mechanism in plants, some special and currently unknown ABA-dependent and ABA-independent signal transduction pathways in cassava exist and need to be further explored.

## 4. Experimental Section

### 4.1. Plant Materials and PEG Treatment

Cassava (*Manihot esculenta Crantz*) variety, KU50, was used in this study. Their stems were cut into approximately 15 cm in length with two to three buds and planted vertically in pots (sand:vermiculite = 1:1; height × upper diameter × bottom diameter = 18.8 cm × 18.5 cm × 14.8 cm). Forty-five days later, plants of similar growth stage (e.g., about 50 cm height and with 6–8 leaves) were selected and subjected to dehydration stress treatments, which were simulated using PEG 6000 solution of different concentrations (20%, 30%, 40% and 50%) at 8:00 a.m., respectively. The plants irrigated with tap water (0% PEG) were considered as control. Leaves were collected at different duration (0, 2, 4, 6, 8, 12, 24, and 48 h after treatment) to measure physiological traits including peroxidase (POD) activity, the content of proline, malondialdehyde (MDA), soluble sugar and soluble protein, and each was repeated three times.

Cassava plants used for RNA-seq sequencing were planted as above, but drought stress was simulated only using 20% PEG this time. Leaves, including folded leaf (FL), full expanded leaf (FEL), and bottom leaf (BL), as well as root (RT) were collected at 0, 3 and 24 h after PEG treatment. Each sample was pooled from 15 plants.

### 4.2. Physiological Traits Determination

POD activity was measured according to Huang, *et al.* [57] with slight modification. About 1 g of leaf sample was grounded in liquid nitrogen with a pre-cooled pestle and mortar, and homogenized in 10 mL extraction buffer containing 0.05 M phosphate buffer (pH 5.5). The homogenate was centrifuged at 4000× *g* for 10 min, and the resulting supernatant was collected and diluted to a final volume of 25 mL for enzyme activity analysis. The assay mixture contained 2.9 mL 0.05 M phosphate buffer (pH 5.5), 1 mL 2% H_2_O_2_, 1 mL 0.05 M guaiacol, and 0.1 mL of enzyme extract. POD activity was determined based on the linear increase in absorbance read at 470 nm for 2 min at room temperature. One unit (U) of POD activity was defined as the increase of absorbance by 0.01 per min, and POD activity was determined as: POD activity (U/g·min) = (Δ*A*_470_ × *V*_T_) × (*W* × *V*_S_ × 0.01 × *t*)^−1^; Δ*A*_470_: alteration of absorbance within 2 min, *W*: weight of leaves (g), *t*: reaction duration (min), *V*_T_: the total volume of extractive enzyme (mL), *V*_S_: the volume of determined enzyme (mL).

MDA content was measured as previously described [58] with minor modifications. 0.5 g of fresh tissue was homogenated in 5 mL of 5% (*w/v*) trichloroacetic acid, and the mixture was centrifuged at 4000× *g* for 10 min at room temperature. Then 2 mL supernatant was mixed with 2 mL 0.67% (*w/v*) 2-thiobarbituric acid and boiled at 100 °C for 30 min, followed by centrifugation at 4000× *g* for 5 min. Absorbance of the supernatant was measured at 450, 532 and 600 nm, respectively. MDA content was calculated with equation: MDA content (μmol/g) = (*C*_MDA_ × *V*_R_ × 10^−3^ × *V*_T_) × (*W* × *V*_S_)^−1^. Of which, *C*_MDA_ = 6.45(*A*_532_ − *A*_600_) − 0.56*A*_450_; *C*_MDA_: MDA concentration (μmol/L); *V*_R_: the volume of reaction (mL); *V*_T_: the volume of total extractive enzyme (mL), *V*_S_: the volume of tested enzyme (mL); *W*: the weight of leaves (g).

Proline content was measured according to Bates, *et al.* [59]. Acid-ninhydrin solution was prepared by warming 1.25 g ninhydrin in 30 mL glacial acetic acid and 20 mL 6 M phosphoric acid at 70 °C with agitation until dissolved, and stored at 4 °C for 2–3 days. Purified proline (Sigma, Ronkonkoma, NY, USA) was used to standardize the procedure for quantifying sample values. The detailed procedures were as follows: (1) approximately 0.5 g of plant material was homogenized in 5 mL of 3% aqueous sulfosalicylic acid and boiled for 10 min, then the mixture was filtered through Waterman paper; (2) 2 mL filtrate was reacted with 2 mL acid-ninhydrin and 2 mL glacial acetic acid in a tube at 100 °C for 0.5 h; (3) 4 mL toluene was added into the reaction mixture and vortexed vigorously for 30 s, then centrifuged 4000× *g* for 5 min; (4) the chromophore containing toluene was aspirated from the aqueous phase, and warmed to room temperature. The absorbance was read at 520 nm using toluene solution for a blank. Proline content was calculated as following: proline content (μg/g) = (*C* × *V*_T_) × (*W* × *V*_S_)^−1^; C: proline quantity calculated by the standard curve (µg); *V*_T_: the volume of total extractive enzyme (mL), *V*_S_: the volume of determined enzyme (mL); *W*: the weight of leaves (g).

Soluble sugar content was measured based on anthrone colorimetric method [60]. The procedures are: (1) cassava leaves were cut into small pieces, then mixed, and approximately 0.3 g was put into 20 mL tube and 10 mL distilled water was added; (2) the samples were heated at 100 °C in a water bath for 30 min and centrifuged at 4000× *g* for 5 min. The supernatant was collected and diluted to a final volume of 25 mL; (3) 0.5 mL extract was added into reaction mixture containing 1.5 mL distilled water, 0.5 mL anthrone ethyl acetate, and 5 mL H_2_SO_4_ in a test tube at 100 °C in water bath for 1 min. Then the reaction was cooled to room temperature and the absorbance was read at 630 nm. Soluble sugar content was determined as follows: soluble sugar content (%) = *C* × (*V*_T_/*V*_S_) × (*W* × 10^6^)^−1^ × 100%.; *C*: sugar quantity determined from the standard curve (µg); *V*_T_: total volume of the extracted solution (mL); *V*_S_: volume of sample solution for testing (mL); *W*: the weight of samples (g).

Soluble protein content was measured by Coomassie Brilliant Blue G-250 staining [60]. The procedures are: (1) 0.5 g of leaf samples were homogenized in 5 mL of distilled water, then centrifuged at 12,000× *g* at room temperature for 10 min. The supernatant was collected as extract; (2) The assay mixture, which contained 1 mL of extract and 5 mL of Coomassie Brilliant Blue G-250 solution, was incubated at room temperature for 2 min. Absorbance was measured at 595 nm, and soluble protein content was calculated with equation: soluble protein content (mg/g) = *C* × *V*_T_ × (*V*_S_ × *W* × 1000)^−1^; *C*: protein quantity determined from the standard curve (µg); *V*_T_: total volume of the extracted solution (mL); *V*_S_: volume of sample solution for testing (mL); *W*: the weight of samples (g).

### 4.3. Library Preparation and Sequencing

Total RNA was extracted using RNA plant reagent kits (Tiangen, Beijing, China). Each RNA-Seq library was constructed as previously described [35]. The libraries were indexed, pooled, and sequenced on Illumina HiSeq2000 ( Illumina, San Diego, CA, USA).

### 4.4. Reads Mapping and Data Analysis

Adapters were removed from raw sequence reads using FASTX-toolkit pipeline version 0.0.13 [61]. Sequence quality was examined using FastQC [62], and low quality reads were filtered also using FASTX-toolkit setting parameters as “q20p80” (*i.e.*, for each read kept, 80% of bases must have sequence quality greater than 20, which indicates 1% sequencing error rate). Clean reads were mapped to cassava genome (version 4.1) obtained from phytozome website [63] using Tophat v2.0.13 [64]. Raw count data were obtained by Cuffdiff embedded in Cufflinks pipeline v2.1.1 [65], and then normalized by library sizes using edgeR [66]. Genes of which max CPM was no less than 10 across the samples were considered as expressed. Similarly to a previous study [36], given two compared conditions, a gene was considered as differentially expressed if it satisfied either of the following two criteria: (1) fold change of CPM no less than 3 if the gene was expressed in both conditions; (2) the gene was not expressed in one condition (CPM < 10) but over-expressed (CPM > 30) in the other condition.

### 4.5. Functional Category Enrichment and Clustering Analysis

Cassava loci were annotated and classified into hierarchical categories based on the MapMan functional classification system [67]. As described previously [68], significantly over-represented functional categories were identified based on Fisher’s exact test or Wilcoxon test that embedded in PageMan [69]. In order to define the changing expression patterns of DE genes across all the samples, hierarchical clustering based on Pearson correlation in MEV program [70] was used to group DE genes. The number of clusters was determined by the FOM (Figures of Merit) method. MEV was also used for gene heat-map visualization.

### 4.6. Quantitative RT-PCR Analysis

RNA-Seq results were verified by quantitative RT-PCR (qRT-PCR) using SYBR-green (TaKaRa Biotechnology Co., Ltd., Dalian, China) and Stratagene Mx3005P system (Stratagene, La Jolla, CA, USA). In total, 15 drought-induced genes, including seven and eight, respectively, located on the ABA-dependent and ABA-independent pathways [37], were selected, and their primers were provided in Table S1 (Supplementary Material). The cassava actin gene [71] was used as an internal control. For each sample, qRT-PCR reaction was repeated three times and the relative mRNA expression level was calculated as 2^−ΔΔ*C*t^. Correlation and significance analyses were performed using Microsoft Office Excel 2007 as described [35].

## Supplementary Materials

Supplementary materials can be found at http://www.mdpi.com/1422-0067/17/3/283/s1.
